# Malocclusion prevalence and orthodontic treatment need in central Anatolian adolescents compared to European and other nations' adolescents

**DOI:** 10.1590/2177-6709.20.6.075-081.oar

**Published:** 2015

**Authors:** Fundagul Bilgic, Ibrahim Erhan Gelgor, Ahmet Arif Celebi

**Affiliations:** 1 Assistant Professor, Mustafa Kemal University, Faculty of Dentistry, Department of Orthodontics, Hatay, Turkey; 2 Professor, Kirikkale University, Faculty of Dentistry, Department of Orthodontics, Kirikkale, Turkey; 3 Lecturer, Ishik University, Faculty of Dentistry, Department of Orthodontics, Erbil, Iraq

**Keywords:** Malocclusion, Orthodontic treatment need, IOTN

## Abstract

**Objective:**

To determine the prevalence of malocclusion and orthodontic treatment need in a large sample of Central Anatolian adolescents and compare them with European-other nations' adolescents.

**Methods:**

The sample included 1125 boys and 1204 girls aged between 12 and 16 years with no previous orthodontic treatment history. Occlusal variables examined were molar relationship, overjet, overbite, crowding, midline diastema, posterior crossbite, and scissors bite. The dental health (DHC) and aesthetic components (AC) of the Index of Orthodontic Treatment Need (IOTN) were used as an assessment measure of the need for orthodontic treatment for the total sample.

**Results:**

The results indicated a high prevalence of Class I (34.9%) and Class II, Division 1 malocclusions (40.0%). Moreover, increased (18%) and reduced bites (14.%), and increased (25.1%) and reversed overjet (10.%) were present in the sample.

**Conclusion:**

Using the DHC of the IOTN, the proportion of subjects estimated to have great and very great treatment need (grades 4 and 5) was 28.%. However, only 16.7% of individuals were in need (grades 8-10) of orthodontic treatment according to the AC.

## Resumo

**Objetivo:**

determinar a prevalência de má oclusão e a necessidade de tratamento ortodôntico em uma amostra extensa de adolescentes da Anatólia Central, e compará-los a adolescentes europeus de outras nacionalidades.

**Métodos:**

a amostra consistiu de 1125 meninos e 1204 meninas, com idades entre 12 e 16 anos, sem histórico de tratamento ortodôntico prévio. As variáveis oclusais avaliadas foram a relação entre molares, *overjet*,*overbite*, apinhamento, diastema na linha média, mordida cruzada posterior e mordida em tesoura. O Componente de Saúde Dental (DHC) e o Componente Estético (AC) do Índice de Necessidade de Tratamento Ortodôntico (IOTN) foram utilizados para avaliar a necessidade de tratamento ortodôntico de toda a amostra.

**Resultados:**

os resultados sugerem uma alta prevalência de má oclusão de Classe I (34,9%) e Classe II, subdivisão 1 (40,0%). Além disso, *overbite*aumentado (18,3%) e reduzido (14,4%), e *overjet* aumentado (25,1%) e reduzido (10,4%) também foram observados na amostra.

**Conclusão:**

com base no uso do DHC do IOTN, observou-se que a proporção dos indivíduos avaliados com necessidade grande e muito grande de tratamento (notas 4 e 5) foi de 28,7%. Porém, de acordo com o AC, apenas 16,7% dos indivíduos apresentaram necessidade (notas 8-10) de tratamento ortodôntico.

## INTRODUCTION

On an increased basis, malocclusion is considered an expression of normal biologic variation, and treatment need is often based as much on psychosocial concerns as on proven oral health risks attributable to malocclusion.^1^ The criteria for determining who is most likely to benefit from orthodontic treatment are controversial. These factors make it particularly difficult for the general dentist to determine for whom orthodontic treatment is clearly indicated, since the traditional pathway to orthodontic care starts at the general dentist's office. 

Different populations have been investigated to provide epidemiological data of the prevalence of malocclusion.^2^^-^ 7 As a common trend, quantitative variables along with Angle's classification were used in these reports. Additionally, treatment-need indexes were also used to determine orthodontic need based on esthetic impairment, potential for adverse effect on dental health, and deviation from normal occlusion.^8^ The Index of Orthodontic Treatment Need (IOTN), involving the Dental Health Component (DHC) and the Aesthetic Component (AC), is the tool most frequently used for measuring treatment need.^9^^,^ 10 Perhaps, being objective and synthetic, and allowing for comparisons between different population groups, are the most important aspects of this index.^7^^,^ 11 ^,^ 12

 Certain European populations, such as the Swedish,^13^ British,^14^ German,^5^^,^ 15 French^16^ and Italian^6^^,^ 7 ^,^ 17 have been examined extensively in regards to IOTN. However, there is little research and/or published data that evaluated together the prevalence of malocclusion^8^^,^ 18 and orthodontic treatment need^19^^,^ 20 in adolescents. Therefore, the aim of the present survey was to document the prevalence of individual traits of malocclusion, and to assess the need for orthodontic treatment in relation to sex by using the IOTN in a group of adolescent schoolchildren. It also aimed to compare the data provided with the findings of chiefly European patients as well as other surveys.

## MATERIAL AND METHODS

Data were collected during an epidemiological survey, in the period of May, 2008 to December, 2012, from 2329 adolescents (1125 males and 1204 females) aged 12.5-16.2 years, randomly selected using a one-stage cluster sampling procedure in 13 state-funded secondary schools in Kirikale city which is located in the south area of the capital of Turkey. The schools were randomly selected from an initial pool of 27 schools that had been previously identified by the school district to avoid possible biases ensuing from social heterogeneity. Written parent informed consent forms were obtained for dental examinations. Family origin and registration information were examined in order to determine that the sample was a good representative of ancestry from the central part of the country. All male and female patients who met the following criteria were included in the sample: (1) age from 12 to 16 years; (2) secondary dentition present with no remaining deciduous teeth; (3) no multiple missing teeth; (4) presence of first permanent canines and molars; and (5) no previous history of orthodontic treatment. Each examination took place while the subject was seated in a standard, quiet classroom in the designated chairs. Clinical examination was carried out by one examiner who was previously calibrated. The examination lasted 20 minutes per child, following the World Health Organization guidelines.^21^

### Orthodontic variables

Patients with an occlusal pattern that deviated from the ideal Class I relationship, which is based on the buccal groove of the mandibular first molar settled on the mesiobuccal cusp of the maxillary first molar as described by Angle, (including crowding, spacing, rotations), were categorized as Class I malocclusion. Thus, the Class I normal category was limited to patients with occlusions that were ideal or near ideal. Patients with a different Angle classification of occlusion on each side were categorized into a single Class based on the predominant pattern of occlusion and/or canine relationship.^4^^,^ 22

For overbite and overjet, values between 0 and 4 mm were considered normal.^7^ Posterior crossbite and scissors bite were registered as bilateral, right and left.^4^^,^ 5 Crowding was recorded for the incisor and also posterior segments of each jaw (1-3 mm = mild; 4-6 mm = moderate; > 6 mm = severe).^4^ Anterior diastema was diagnosed when there was a space of at least 1 mm between central incisors in either arch.^4^

Patients with a normal occlusion pattern had normal molar and canine relationships, no crowding or crossbites, normal overjet and overbite, well-balanced faces, and no history of previous orthodontic treatment.

### Orthodontic treatment need

The findings served to determine orthodontic treatment need with reference to the IOTN^9^^,^ 10 which consists of the DHC and the AC. Considerations as to no treatment need, borderline need, or great need were based on five grades in the DHC and 10 grades in the AC.

### Statistical analysis

To test examiner reproducibility, 25 children were reexamined by Kappa's method four weeks after initial examination.^23^The ratio of the sample, as a maximum estimate of the proportion of individual traits of malocclusion in the whole population, was calculated for the total sample and for girls and boys separately. The number of subjects with diagnosed anomaly (n) and its prevalence (n/N x 100, in which N is the number of subjects examined) was determined. The differences between sex groups were assessed by means of chi-square test. Data were analyzed by SPSS software package (version 21.0, SPSS Inc., Chicago, Ill., USA). for IOTN DHC and AC grades. Level of significance was established at *p* < 0.05.

## RESULTS

Kappa test indicated high reliability and reproducibility (0.73 - 0.80) for the parameters tested. Table 1 presents the prevalence of each occlusal trait in the total sample. Class I malocclusion was found in 812 subjects, which represented 34.9% of the 2329 individuals examined. Class II malocclusion was diagnosed in 1041 individuals, 40.0% of all patients were Division 1 and 4.7% of all patients were Division 2. Class III malocclusion was found in 240 subjects, 10.3% of the sample. Normal overbite was the most common (73.5%), mostly observed in girls (*p* < 0.001). Increased overbite was recorded in 18.3% of the sample, mostly observed in boys (*p* < 0.05). The prevalence of reduced bite value was found as 8.2%. Normal overjet was present in 1371 individuals (64.5%). Prevalence of increased overjet (25.1%) was found to be higher than negative overjet (10.4%). While crossbite was found more frequently, as much as of 4.0 % of the sample, scissors bite was rarely diagnosed in only 0.3% of the subjects.

**Table 1:** - Occlusal classifications.

		**Boys**	**Girls**	**Total**	***p*-value**					
			**n**	**%**	**n**	**%**	**n**	**%**		
Occlusal anteroposterior relationships										
Normal occlusion			110	9.8	126	10.5	236	10.1	NS	0.63
Class I			404	35.9	408	33.9	812	34.9	NS	0.317
Class II, Division 1			448	39.8	483	40.1	931	40.0	NS	0.899
Class II, Division 2			56	5.0	54	4.5	110	4.7	NS	0.625
Class III			107	9.5	133	11.0	240	10.3	NS	0.246
Distribution of overbite										
Normal (0-4 mm)			802	71.2	913	75.8	1715	73.5	***	0.0001
Increased (> 4 mm)			227	20.2	197	16.4	424	18.3	*	0.018
Reduced (< 0 mm)			96	8.5	94	7.8	190	8.2	NS	0.098
Distribution of overjet										
Normal			731	65	770	64	1501	64.5	NS	0.866
Increased			281	25	304	25.2	585	25.1	NS	0.886
Negative			113	10	130	10.8	243	10.4	NS	0.588
Distribution of posterior crossbite and scissors bite										
	No finding		1021	90.8	1082	89.9	2103	90.3	NS	0.677
Crossbite	Bilateral		41	3.6	52	4.3	93	4.0	NS	0.544
	Unilateral	right	35	3.1	41	3.4	76	3.3	NS	0.890
		left	24	2.1	27	2.2	51	2.2	NS	0.970
Scissors bite	Bilateral		1	0.1	1	0.1	2	0.1	NS	0.957
	Unilateral	right	2	0.2	0	0.0	2	0.1	NS	0.949
		left	1	0.1	1	0.1	2	0.1	NS	0.889

NS: Not significant. *p < 0.05; ***p < 0.001.

Anterior crowding was present in 1638 individuals (66.2%) (Table 2); 17.9, 9.1 and 38.1% of those had crowding in the upper arch, lower arch and both arches, respectively. Moderate crowding was more common in both arches. Midline and spread diastemas were found in 14.8 and 12.9% of the sample, respectively. Diastemas were observed mostly in the upper arch (Table 2).

**Table 2:** - Distribution of crowding and diastema.

		**Boys**	**Girls**	**Total**	***p***				
		**n**	**%**	**n**	**%**	**n**	**%**		
Crowding									
No crowding		383	34.0	428	35.5	811	34.8	NS	0.460
Upper arch, only	mild	140	12.4	120	10.0	260	11.2	NS	0.214
	moderate	55	4.9	60	5.0	115	4.9	NS	0.732
	severe	18	1.6	24	2.0	42	1.8	NS	0.810
Lower arch, only	mild	67	6.0	70	5.8	137	5.9	NS	0.845
	moderate	28	2.5	31	2.6	59	2.5	NS	0.760
	severe	8	0.7	9	0.7	17	0.7	NS	0.77
Both arches	mild	280	24.9	303	25.2	583	25.0	NS	0.981
	moderate	127	11.3	137	11.4	264	11.3	NS	0.831
	severe	19	1.7	22	1.8	41	1.8	NS	0.985
Diastema									
No finding		808	71.8	852	70.8	1660	71.3	NS	0.328
Upper arch	midline	140	12.4	156	13	296	12.7	NS	0.214
	spread	99	8.8	95	7.9	194	8.4	NS	0.632
Lower arch	midline	20	1.8	30	2.4	50	2.1	NS	0.870
	spread	58	5.2	71	5.9	129	5.5	NS	0.670

NS: Not significant.

**Figure 1:**
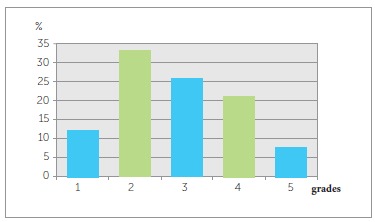
- Dental Health Component (DHC) grades of the Index of Orthodontic Treatment Need (IOTN) in Anatolian adolescents (Grades 1 and 2, no need; Grade 3, borderline need; Grades 4 and 5, definite need).

**Figure 2:**
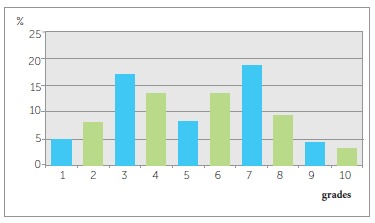
- Aesthetic component (AC) grades of the Index of Orthodontic Treatment Need (IOTN) in Anatolian adolescents (Grades 1-4, no need; Grade 5-7, borderline need; Grades 8-10, definite need).

In the study group, the IOTN revealed no treatment need in 45.6% of the sample, when the DHC was used (mostly in boys (*p* < 0.05) and 43.1% when the AC was used. (Figs 1 and 2, and Tables 3 and4). When borderline cases were taken into consideration, treatment need was diagnosed in 25.7% of the sample when the DHC was used and in 40.2% when the AC was used. The number of subjects with the need for orthodontic treatment was 648 (28.7%) when the DHC was used, and 376 (16.7%) when the AC was used. 

**Table 3:** - DHC of IOTN statistics of boys and girls.

**Occlusal anteroposterior relations-hips**	**Boys**	**Girls**	**Total**		***p*-value**			
	**n**	**%**	**n**	**%**	**n**	**%**		
No need	531	48.3	492	42.8	1023	45.6	*	0.01
Borderline need	263	23.9	316	27.5	579	25.7	NS	0.054
Need	306	27.8	342	29.7	648	28.7	NS	0.328
Total	1100	100	1150	100	2250	100		

*,*p* < 0.05; NS: Not significant.

**Table 4:** - AC of IOTN statistics of boys and girls.

**Occlusal anteroposterior relationships**	**Boys**	**Girls**	**Total**		***p*-value**			
	**n**	**%**	**n**	**%**	**n**	**%**		
No need	492	44.6	478	41.6	970	43.1	NS	0.136
Borderline need	437	39.6	467	40.7	904	40.2	NS	0.699
Need	171	15.4	205	17.9	376	16.7	NS	0.142
Total	1100	100	1150	100	2250	100		

NS: Not significant.

## DISCUSSION

Although many studies were published to describe the prevalence and types of malocclusion, when examining a certain population it is difficult to compare and contrast these findings, partly because of the varying methods and indexes used to assess and record occlusal relationships, age differences of the study populations, examiner subjectivity, specific objectives, and differing sample sizes.^22^ Methodology used in this study was mostly collected from European studies,^4^^,^ 6 ^,^ 7 ^,^ 22 and our results were discussed with the findings from different European geological regions due to close proximity and since there was limited information of individuals in the literature. The general consensus about treatment timing for malocclusions is that it should start around permanent dentition. At this stage, maxillary and mandibular development is almost completed and the malocclusion takes its final pattern. Given the characteristics of the sample, this paper demonstrated the occlusal traits of an untreated adolescent population at those ages. 

With respect to the occlusal findings, Class I malocclusion was found in 34.9% of the sample. This Class I occlusion figure included individuals with incisor crowding and dental malalignment and thus did not imply ideal normal occlusion. The prevalence of Class II, Division 1 (40.0%), in the present study, was greater than the rates reported for English school children (12.5%),^24^ Shropshire school population (27.2%),^25^ adolescents in Bogotá (14.9%),^4^ and Italian school adolescents (36.3%.^7^ However, Lauc^26^ on Hvar Island, and Josefsson et al^13^ for a Swedish population, found that Class II malocclusion was more common in their population (greater than 45%), and explained this figure by a genetic influence on the incidence of Class II malocclusions. Early treatment in the primary or early mixed dentition has been recommended for Class III malocclusions.^4^^,^ 27 The prevalence of Class III malocclusion determined in this study is 10.3%. However, Goose et al^28^ (2.91%), Haynes^24^(2.5%), Foster and Day^25^ (3.5%), Proffit et al^29^ (5.7%), Thilander et al^4^ (5.8%), Lauc^26^ (4.8%), and Perillo et al^7^(4.3%) reported lower rates. The present study confirmed that the predominant anteroposterior relationship of the arches in adolescents was Class II, Division 1. Of the vertical anomalies, increased overbite was more than twice as frequent as anterior open bite. Our results were similar to the rates reported by Thilander et al^4^ and Lauc^26^ who also claimed that deep bite was often associated with a Class II malocclusion and more common in boys. However, higher ratios were found in Italian samples.^6^^,^ 7 Increased overjet proved to be as high as increased overbite in this study; this is a reflection of the higher prevalence of Class II malocclusion among adolescents. Our findings agree with those of Thilander et al,^4^ in Bogotanian adolescents, and Ciuffolo et al,^6^ in Italian adolescents, in which high rates of increased overjet in the permanent dentition were reported. In a French sample, increased overjet was present in fewer subjects (6%).^3^

In this study, uni/bilateral posterior crossbite was more frequent than scissors bite and was observed in 9.5% of the sample. This rate was similar to the findings of Ciuffolo et al^6^ and higher than Thilander et al.^4^ Perillo et al^7^ showed a higher percentage for crossbite and scissors bite (14.2 and 3.5%, respectively).

Crowding, in one or both arches, was the most frequent of all anomalies recorded (66.2%). This finding complied with the results of Thilander et al^4^ and Lauc.^26^ There is a general consensus that treatment of crowding should start in the permanent dentition.^5^ The National Health and Nutrition Survey III, undertaken in the United States between 1989 and 1994, showed a frequency of crowding ranging from 42.3% at ages 8-11 to 54.5% at ages 12-17, which was lower than the frequencies observed in this investigation.^29^ Nevertheless, other studies have reported lower rates of crowding located in anterior/both segments.^3^^,^ 6 ^,^ 7 ^,^ 24 ^,^ 25

Thilander et al^4^ found the prevalence of median diastema in their population to be 13.5% in the early mixed and 4% in the permanent dentition. Lauc^26^ observed a high rate of midline diastema (45.1%). In contrast, in our study, this rate was 12.7%. Perillo et al^7^ showed the prevalence of median diastema as equal to 9.9%. The frequency of diastema in Nigeria was 24%.^30^

Administrators of publicly funded programs need a valid screening method to determine priority for orthodontic treatment.^15^Priority of orthodontic care through national health care plans in European countries has been a prime factor behind the development of indexes, such as the IOTN. 

The need for orthodontic treatment has been presented in the literature by means of different indexes. In the present study, the classification by the IOTN was used because the authors' are familiar with this index. 

In Turkey, there are few epidemiologic surveys. Guray et al^19^ used the Treatment Priority Index (TPI) and found that 72.26 % of 483 students required orthodontic treatment in a primary school with a low socioeconomic standard from Konya district (South Anatolia). Ugur et al^20^ found a 37.77% orthodontic treatment need, by using the TPI in 572 6 to 10-year-old Turkish primary school children with a high socioeconomic standard in central Anatolia. Our study was carried out in a large adolescent sample with moderate socioeconomic status, and treatment need was lower than those two studies. These studies conducted in different regions show similar results in terms of the need for orthodontic treatment in individuals with different socio-cultural features in different locations. The results of this study were not in agreement with Ugur et al^20^ who determined that orthodontic treatment needs increase with age. In our study, according to the DHC of the IOTN, 28.7% of the whole sample was classified as being in need of orthodontic treatment (grades 4 and 5). The results showed that the percentage was relatively greater than those reported by Souames et al^16^ in France and Perillo et al^7^ in Italy (21.3 and 27.3%, respectively). However, the British studies found a higher prevalence rate for untreated subjects: 32.7%,^10^ 33% and, 35%.^14^Josefsson et al^13^ found 39.5% of orthodontic treatment need in a Swedish sample. The findings of the present study, therefore, indicated that a substantial need for orthodontic intervention was present at a similar level to French and Italian children, but generally lower than northern European populations (United Kingdom and Sweden). 

The AC for IOTN, in the present study, reduced orthodontic treatment need (16.7%). This has also been reported in other studies.^10^^,^ 13 ^,^ 16 Tausche et al^5^ claimed that the AC alone failed to identify any children needing orthodontic treatment. Because of the AC alone is an inappropriate method for screening treatment need, lack of agreement occurs between the normative component and the IOTN-AC. However, Josefsson et al^13^ used the AC both by the examiner and the subject. This study also hunted up a difference between males and females for orthodontic treatment need. Treatment need did not differ significantly as a result of sex. AC alone is unsuitable for screening treatment need.

## CONCLUSION

The results of this investigation demonstrated that Class II, Division 1 malocclusion was the most prevalent occlusal pattern among adolescents, and the high incidence of increased overjet and overbite are a reflection of the high prevalence of Class II malocclusion. Also, a high percentage of crowding is noteworthy. Nearly one-third of the evaluated population would have a mandatory need for orthodontic treatment, if the DHC scores were used as the main criterion for such decisions. If the AC scores were used, the need would decrease to one-fifth of the sample. These results revealed the high percentage of need for orthodontic treatment in Turkey.
